# Evaluation of the quality of fit of flexible bolus material created using 3D printing technology

**DOI:** 10.1002/acm2.13490

**Published:** 2022-01-20

**Authors:** Ciaran Malone, Elaine Gill, Tanith Lott, Catherine Rogerson, Sinead Keogh, Majed Mousli, Denise Carroll, Caitriona Kelly, John Gaffney, Brendan McClean

**Affiliations:** ^1^ St. Luke's Radiation Oncology Network Dublin, Ireland

**Keywords:** 3D printing, bolus, clinical application, radiotherapy

## Abstract

**Aims:**

To retrospectively evaluate the quality of fit of 3D printed bolus over four different treatment sites to determine whether certain sites favor a 3D printed approach and if the quality of fit changes over the course of treatment.

**Materials and methods:**

A retrospective analysis of the first 60 cases treated using 3D printed bolus in our radiotherapy center was undertaken. All boluses were printed using flexible thermoplastic polyurethane (TPU) material. We developed a system of rating the quality of fit using four quality categories. The analysis of 60 patients consisted of a review of a total 627 treatment fractions for head and neck (H&N), scalp, pelvis, and extremity treatment sites.

**Results:**

Out of 627 fractions evaluated, 75.1% were rated either “good” or “excellent”, 20.6% were rated as “acceptable” and 4.3% were rated “poor”. H&N, scalp, and extremity treatment regions were found to favor a 3D printed approach. However, pelvis cases had a higher proportion of “acceptable” and “poor” ratings. Trend analysis showed no notable change in the quality of 3D printed bolus fit over the course of treatment, except for pelvis cases which tended to change categories more than other treatment sites.

**Conclusion:**

This evaluation demonstrates that 3D printed bolus, created using semi‐flexible materials such as TPU, is an effective and practical bolus choice for radiotherapy. In particular, using a 3D printed approach for H&N, scalp, and extremities was found to have a highly conformal fit.

## 1 INTRODUCTION

Bolus is commonly used for both photon and electron radiotherapy to increase the dose to superficial tissue. Ideally, bolus should conform to the patient surface, be approximately tissue equivalent, comfortable, reproducible, easy to place day‐to‐day, and flexible to account for changing patient anatomy throughout treatment. There are many different types of bolus available, each with their inherent advantages and disadvantages.^1^ Traditionally, sheets of uniform thickness bolus, typically 5 or 10 mm, are used to ensure the prescribed dose is delivered to the superficial target volume. However, using flat bolus sheets on an irregular or curved patient surface results in air gaps degrading the build‐up effect.^2^, ^3^, ^4^, ^5^, ^6^ Alternative solutions include moldable boluses, such as wax,^7^, ^8^ thermoplastic sheets^7^, ^9^ or thermoplastic pellets,^7^ which can be difficult and labor intensive to mold accurately to the desired thickness. Wet gauze, which is also highly user‐dependent, is difficult to place and can vary from the assigned treatment plan density considerably.^2^

Recent advancements in 3D printer technology and materials allow the creation of radiologically water equivalent bolus materials that can conform to patient anatomy. A commercial solution by 3DBolus (Adaptiiv Inc., Halifax, NS, Canada) integrates the design and creation of 3D printed bolus directly into the radiotherapy workflow. 3DBolus software converts the bolus generated by the treatment planning system to a file suitable for 3D printing. Thermoplastic polyurethane (TPU) was used as our printing material of choice. It offers improved flexibility compared to the rigid materials described in previous studies.^10^, ^11^, ^12^, ^13^, ^14^, ^15^ The resultant bolus is bespoke to the patient's surface anatomy offering the potential for improved conformality, comfort and ease of placement throughout the patient's treatment, compared to traditional bolus approaches. However, to our knowledge only individual case studies or studies with small sample sizes that assess the benefit of using 3D printed bolus have been presented.^10^, ^12^, ^13^, ^14^, ^16^, ^17^, ^18^, ^19^ In this study, we present the results of 60 patients that were treated with 3D printed bolus in four different anatomical regions: head and neck (H&N), pelvis, scalp, and extremities. The aim was to assess the quality of fit of 3D printed bolus materials, whether certain sites are more favorable to a 3D printed approach than others and monitor the quality of the fit changes over the course of treatment.

## 2 METHODS

### 2.1 Objectives

1. Evaluate the overall quality of fit of 3D printed bolus.
2. Assess the suitability of treatment sites to a 3D printed approach.
3. Evaluate if the quality of fit changes over the course of treatment.

### 2.2 Organization of the study

The evaluation group consisted of seven experienced staff members including physicists, radiation therapists, and radiation oncologists. It was decided to include our four most common anatomical regions to ensure adequate numbers in the analysis. The quality of fit was grouped into four distinct categories to ensure each reviewer was given the same guidance, outlined in Table 1. Each treatment fraction was evaluated by at least two different people. For each treatment fraction the fit of the bolus was evaluated using cone‐beam computed tomography (CBCT) on all three planes (axial, sagittal, and coronal) with the results entered into a standardized form. The reviewer chooses a suitable “fit” category for each fraction using Table 1 as a guide. If air gaps ranged between 1 and 5 mm a rating of “good” was given, if any air gaps ranged between 5 and 10 mm were present, a rating of “acceptable” was given. The <5, 5–10, and >10 mm air gap criteria for categorizing the bolus fit for each treatment fraction, shown in Table 1, was based on a literature review of air gaps using bolus for photon radiotherapy. Butson et al.^6^ showed that air gaps less than 10 mm resulted in an acceptable reduction of skin dose and, on all occasions, more than 90% of maximum dose is still applied to all skin regions. Similarly, Chung et al.^20^ found up to 10% differences on surface doses for 10 mm air gap when they used 10 × 10 cm^2^ field size and 60° angle of incidence. Boman et al.^21^ found that for Volumetric Modulated Arc Therapy (VMAT) techniques the presence of 10 mm air gaps reduced the surface dose on average by 13.6%. Rustgi et al.^22^ showed that for a 25 mm diameter field and air gap thicknesses of 3, 4.6, 6, and 9.2 mm, the corresponding dose reduction is 3%, 4%, 7%, and 13%, respectively. These data were used to form the four categories shown in Table 1. After the data collection, a statistical analysis on the data was carried out.

**TABLE 1:** Classification guidance for the evaluation of bolus fit

Type of air gap	Excellent fit: no notable air gap	Good fit: <5 mm	Tolerable fit: 5–10 mm	Poor fit: >10 mm
Description	No gaps of significance noted	Some minor air gaps, clinically acceptable, no action required	Some small areas up to 5–10 mm, clinically acceptable but action required if consistent	Significant gaps throughout, some regions >10 mm. Not acceptable, action required

### 2.3 Summary of patient data

The first 60 patients treated using a 3D bolus approach were used in this analysis. Only patients with CBCT imaging were included in this study. Unless there were issues with coverage or significant setup errors, the Extended No Action Limit (e‐NAL) IGRT strategy as per the Royal College of Radiologist's On Target^23^ guidance document was followed for all patients. The e‐NAL IGRT strategy requires that a CBCT is taken on fractions 1–3, moves verified on fraction 4 followed by weekly CBCT for the remainder of treatment. The average number of CBCTs acquired per patient was 11# +/‐ 9# resulting in a total of 627 imaging fractions to be analyzed. We examined four different treatment sites: H&N, scalp, extremity, and pelvis, as these four sites were our most frequent sites requiring bolus. Scalp boluses were classified as any bolus that covered the crown of the head. We decided to analyze scalp bolus separately to H&N as the computed tomography (CT) slice thickness (2.5 mm) was deemed to potentially affect the superior–inferior accuracy of the printed bolus around the crown of the head.

### 2.4 Bolus generation and printing

To generate a 3D printed bolus that conforms to the patient surface, the body contour in the treatment planning system must accurately represent the real patient surface. Each bolus was generated using the bolus generation and contouring tools available in Eclipse (Varian Medical Systems Inc., Palo Alto, CA, USA). For cases where bolus was required to fit around complex external contours (e.g., H&N and extremity cases) a 1–2 mm uniform expansion of the body contour before bolus generation was undertaken in eclipse as standard. The bolus is then checked by a physicist to ensure the bolus represents the patient's body contour, the bolus has no “holes” or features that are unsuitable for printing and the bolus has a suitable surface to place as the first layer on the printer bed. The bolus contour was then exported, processed and converted to an Stereolithography file suitable for printing using 3DBolus.

Three‐dimensional printing was undertaken using an AirWolf Axiom 20 3D printer (Airwolf3D, Las Vegas, NV, USA) with TPU filament (either WolfBend,^24^ Cheetah,^25^ or RS PRO^26^). All three TPU types were found to be dosimetrically equivalent with comparable properties. Each bolus was printed at 30 mm/s using a 0.5 mm nozzle, a nozzle temperature of 240°C, a layer height of 0.3, and 100% fill selected. One hundred percent infill was used to obtain a “fully solid” print that was as homogeneous as possible. Each bolus was CT scanned after printing to verify homogeneity and bolus density.

### 2.5 Clinical cases

Out of a total of 60 cases, eight cases were chosen as examples (Figures 4, 5, 6, 7) representing each of the different rating categories from our H&N, scalp, pelvis, and extremity cohorts. Each case selected had complete agreement on the quality of fit from all reviewers.

## 3 RESULTS

### 3.1 Quality of fit using 3D printed bolus

**FIGURE 1:**
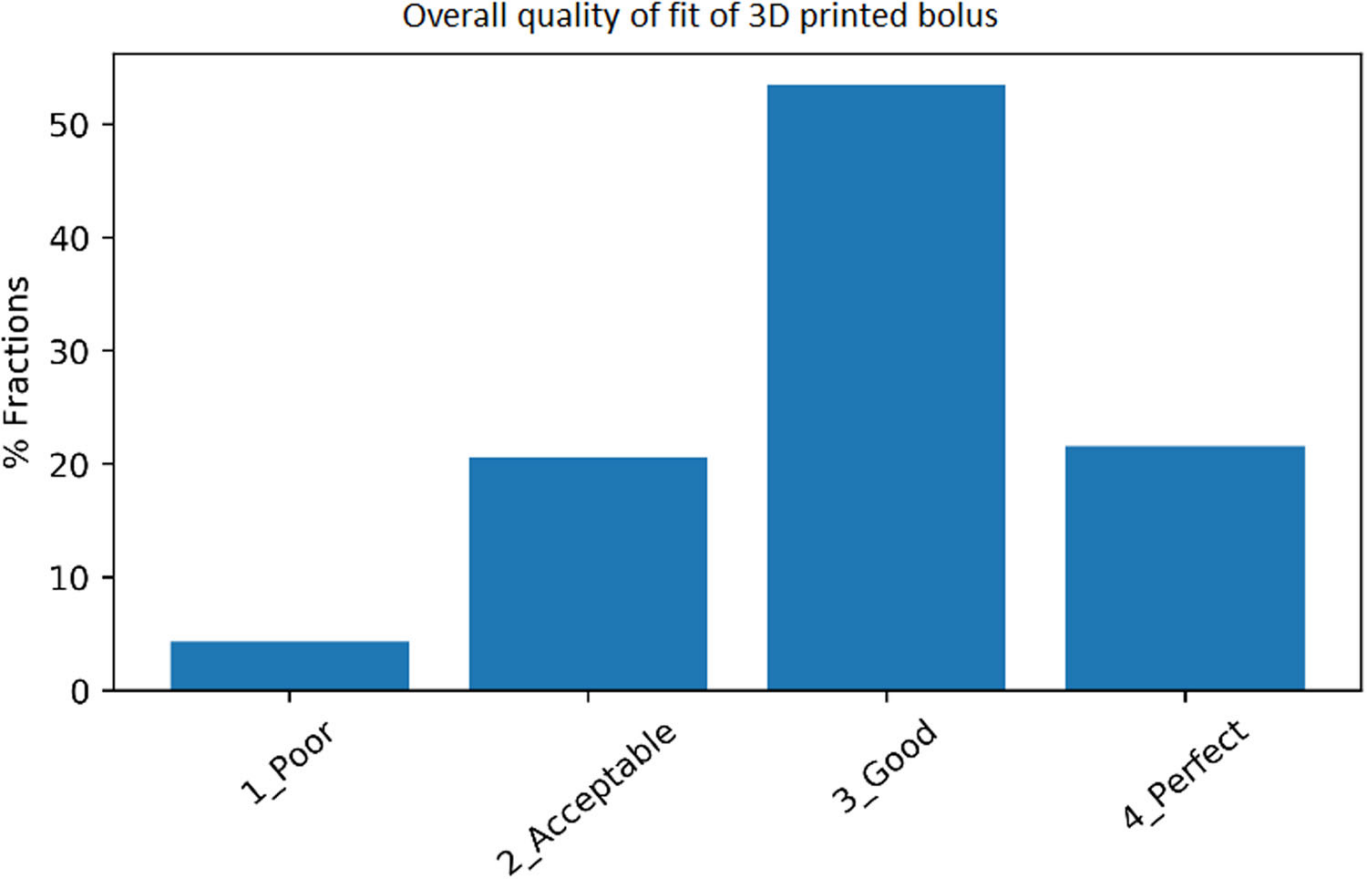
Distribution of 3D printed bolus ratings for all sites

**FIGURE 2:**
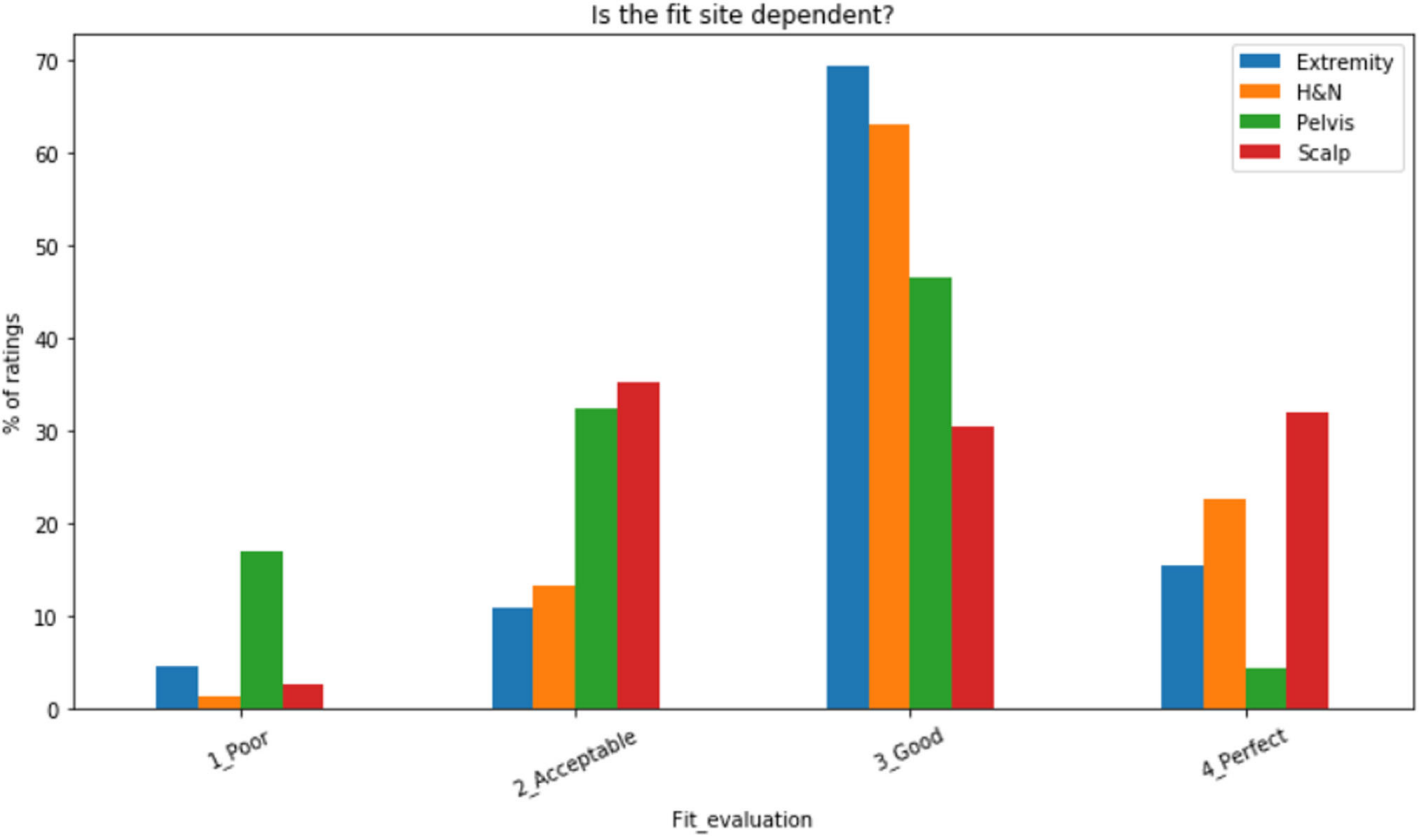
How each treatment sites ratings were distributed. Each site's rating across the four categories sums to 100%

Results in Figure 1 show that out of 627 fractions evaluated, 75.1% of fractions were rated either “good” or “excellent”, 20.6% were rated as “acceptable”, and 4.3% were rated “poor”. Figure 2 normalizes the ratings by site and the proportion of each site's ratings that fall into each category are shown. The highest rate of “poor” ratings were in the pelvic region with 20% of all pelvic fractions receiving a “poor” rating compared to 1% of H&N fractions. H&N and scalp regions had the highest proportion of “excellent” ratings, 22% and 30%, respectively, compared to 3% of pelvis fractions. This suggests that 3D printed bolus using TPU type materials is more suited to the H&N and scalp regions and does not perform well in the pelvic region.

### 3.2 Does the quality of fit changes over the course of treatment?

**FIGURE 3:**
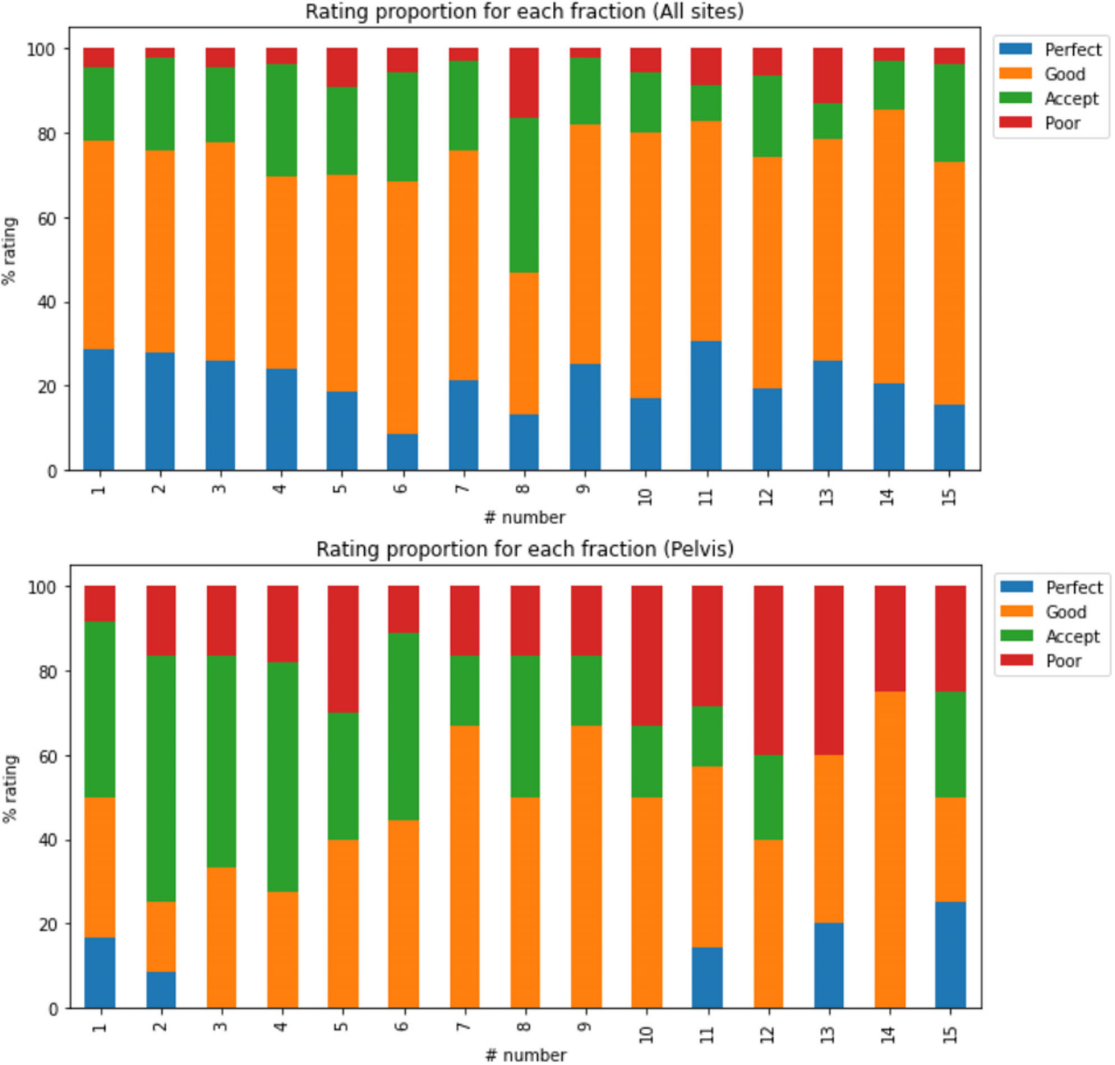
Proportion of each rating, on a per fraction basis, over the course of treatment. Top: all clinical site data; bottom: pelvis data only

Results showing the quality of fit for each individual fraction can be seen for all sites in Figure 3 (top) and pelvis only in Figure 3 (bottom). Presenting each fraction in this way highlights how the proportion of ratings change over the course of treatment. As few patients were treated with a total number of fractions >20, the number of fractions displayed is limited to 15# to ensure at least 10 independent data points were present for each fraction. No clear change in the quality of bolus fit was found across all sites as the treatment progressed. Only the pelvis data showed some apparent change with the number of “poor” ratings increasing as the treatment progressed. Evaluating this further, the increase in the number of poor ratings mostly arose from “acceptable” cases that deteriorated to “poor”. It should be noted that a comparable proportion also moved to the “good” category suggesting the fit tends to change over the course of treatment for pelvic cases.

### 3.3 Limitations of the study

Most of the patients treated with bolus were palliative in nature, this resulted in a notable difference in the number ratings for fractions between 1 and 15 compared to 15+. Therefore, our evaluation of how fit changes over the course of treatment was limited to patients with ≤15 fractions as only a small proportion of patients (10 patients) had fractionation schedules >15# in this study. This resulted in us only being able to assess if there were variations over the course of treatment up to fraction 15.

Each treatment fraction was rated by at least two different people to help reduce reviewer bias. Over 80% of all fractions were in agreement, 20% were rated with a category difference of 1, and 3% were rated with a category difference of 2. Of note, a category difference of two corresponds to one reviewer rating “excellent” and another rating “poor” for the same treatment fraction. Fractions with a difference in rating of 2 were evaluated by an group independent of the initial evaluation comprising of two radiation therapists to make a final decision on the appropriate rating.

### 3.4 Clinical results

#### 3.4.1 Pelvis cases: squamous cell carcinoma of the vulva

**FIGURE 4:**
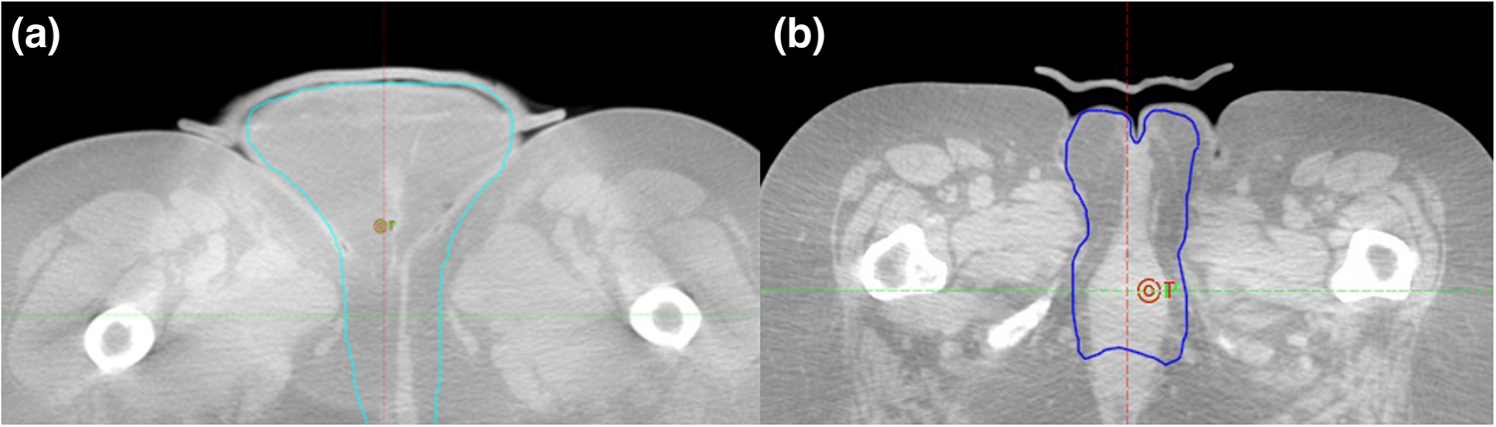
Two vulva SCC cases treated using 3D printed bolus. Excellent rated treatment fraction (a); poor rated treatment fraction (b)

Traditionally, wet gauze would be used in our center to pack the vulva region ensuring both a comfortable and conformal fit. However, wet gauze bolus can be very variable depending on how it is made.^2^ To avoid a potential decrease in dosimetric coverage using wet gauze, a 3D printed bolus was used to achieve both a consistent placement and density during treatment. Figure 4 shows both an “excellent” and a “poor” rating for the position of 3D printed bolus. However, the quality of fit for both cases was found to be variable over the course of treatment.

#### 3.4.2 Scalp cases: basal cell carcinoma of the scalp

**FIGURE 5:**
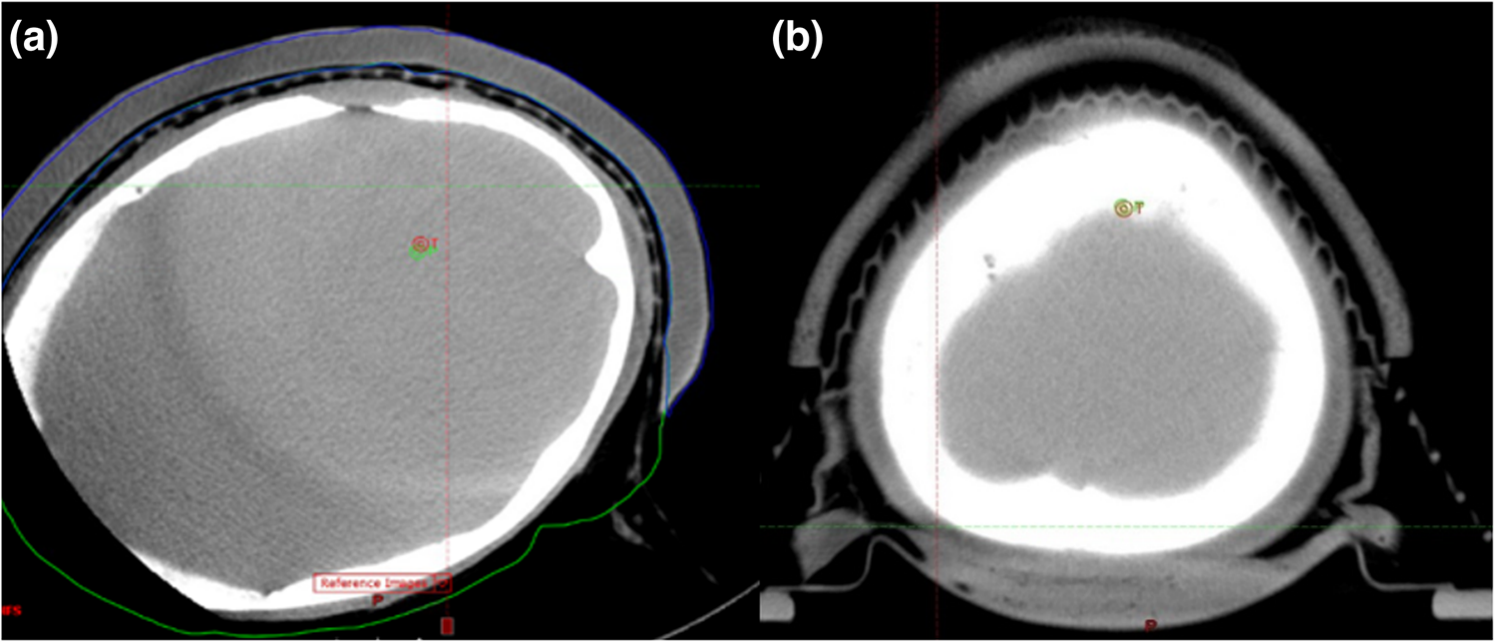
Extensive BCC of the scalp. Excellent rated fraction (a); acceptable rated treatment fraction (b)

Figure 5 highlights two cases where a 3D printed bolus approach was used for the scalp. Figure 5a is an example of a case which was predominantly rated “excellent” for each fraction. This patient was originally not for 3D printed bolus. Due to the sensitivity of the skin under the bolus, using thermoplastic bolus resulted in discomfort due to the uneven distribution of the weight across the scalp. Using multiple sheets of Superflab resulted in air gaps and significant time to reproduce day‐to‐day. A 3D printed bolus approach was attempted and resulted in a comfortable fit due to the weight being evenly distributed across the scalp. Figure 5b shows a fraction of a different patient whose bolus fit was rated “acceptable”. The bolus was not found to be flush with the surface of the mask on several fractions. Review of the bolus fit found that the bolus generated in the planning system overlapped with the treatment mask in one location, causing it to “pitch” up when taped to the thermoplastic mask on‐set.

#### 3.4.3 Extremities: Lymphoma

**FIGURE 6:**
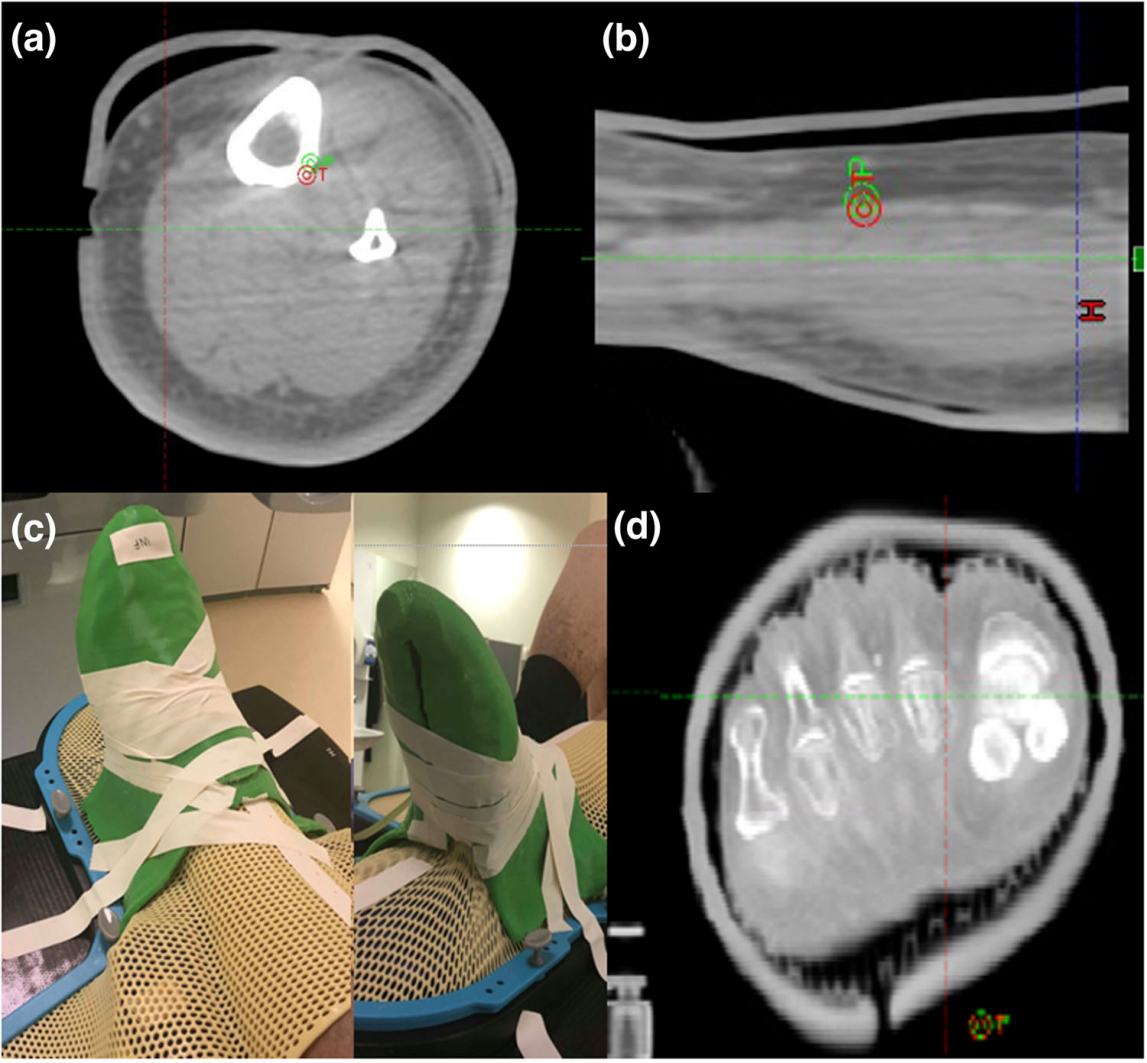
Examples of the 3D bolus fit on extremity patients. “Acceptable” rated fraction for leg bolus case (a and b). “Excellent” rated fraction for foot and ankle case (d), with corresponding photo of setup showing the generated slit to allow for swelling (c)

Figure 6 shows two extremity patients treated with 3D printed bolus. The first required 0.5 cm bolus for their entire foot and ankle and the second required 0.5 cm bolus for their entire lower leg. Extremity treatment setup times using bolus are typically long and laborious, potentially resulting in inconsistent coverage due to the difficulties in conforming flat sheets of bolus to the skin surface. For these reasons, a 3D printed bolus approach was undertaken. Although the time required to 3D print a bolus is typically far greater than a typical CT simulation session requiring extremity bolus, the patient is not required to be present during the 3D printing. This results in a much shorter CT simulation session. Each 3D printed bolus was generated with a slit along the superior–inferior direction using eclipse contouring tools to allow for adjustment using leucoplast tape to pull the slit closed when a tighter fit was required. This ensured that each bolus was easily adapted to account for any edema that occurred during treatment. Both patients bolus fit well throughout treatment (either “good” or “excellent” ratings for each fraction). However, the full leg wrap experienced a single “acceptable” rated fraction which is shown in Figure 6a. Due to the sensitivity of the skin and the extent of the leg being treated, it was difficult to ensure the rotation and fit was good before imaging. We recommend using 3D printing software to cut the bolus into two separate pieces before printing for full arm/leg wraps for any future cases to ensure ease of positioning and placement.

#### 3.4.4 H&N cases: SCC of the tragus and skin

**FIGURE 7:**
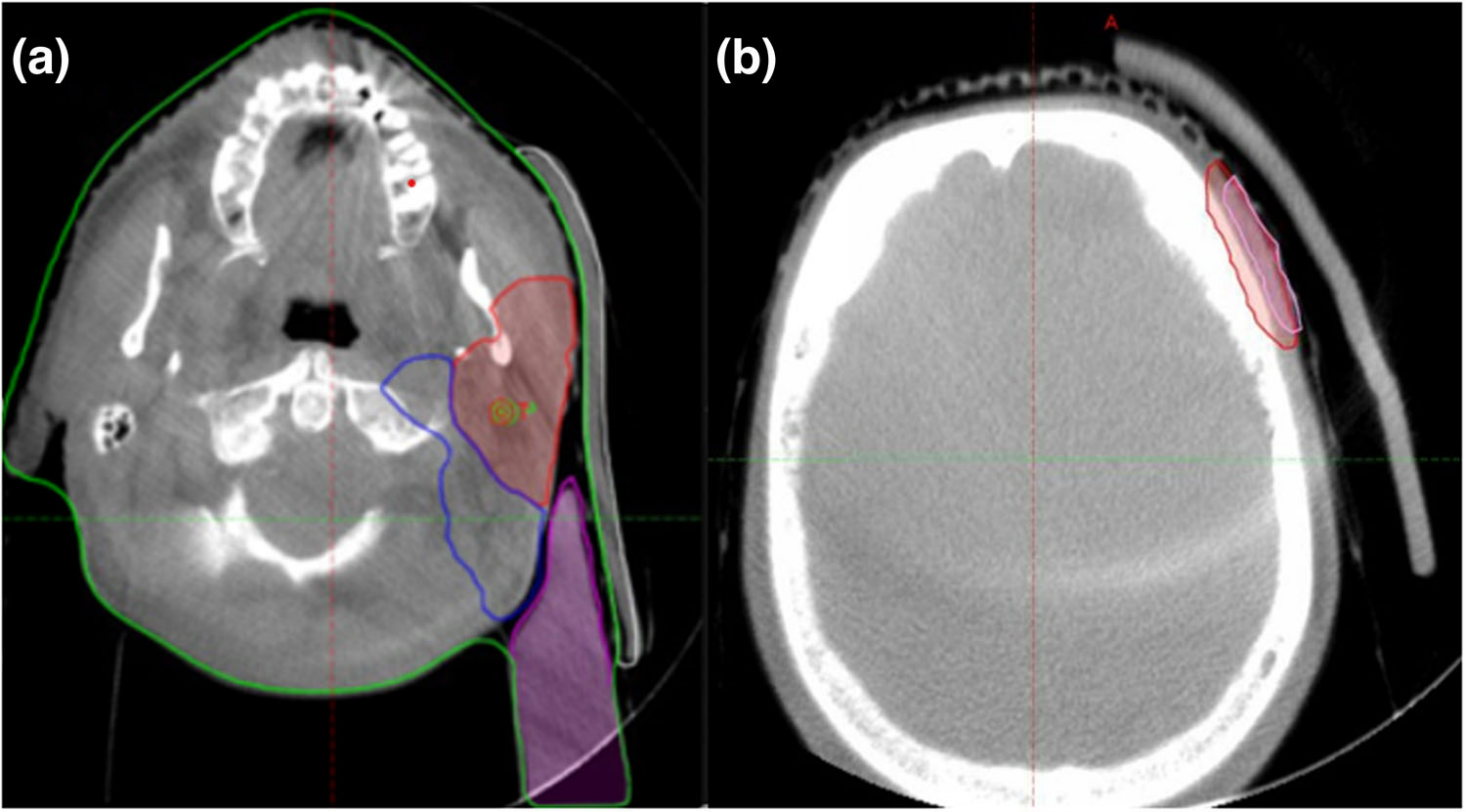
Examples of the 3D bolus fit on head and neck (H&N) patients. “Excellent” rated treatment fraction (a); “poor” rated treatment fraction (b)

Figure 7 demonstrates an “excellent” and “poor” rated 3D bolus H&N fraction. The patient in Figure 7a is presented with a left‐sided moderately differentiated SCC of the tragus following resection. As the immobilization mask is fixed at points on the treatment couch away from the body, there is a significant gap between the patient surface and mask at the posterior aspects of the treatment volume. Due to this gap, two bolus pieces were required, one outside the mask and one inside the immobilization mask. The bolus was contoured to fit in the cavity and conform to the fixation screws present to hold the headrest in place. The outside bolus was also 3D printed to ensure precise placement with the desired amount of overlap. Figure 7b shows a similar setup, without the bolus inside the mask, where the 3D bolus was rated as “poor”. The issue was remedied with extra leucoplast tape affixing the bolus to the thermoplastic shell to prevent the bolus sliding down due to gravity, which tended to improve the bolus rating to “good” for the majority of the remaining fractions.

## 4 DISCUSSION

Bolus is commonly used in the radiotherapy workflow and has remained largely unchanged from the sheets of uniform thickness bolus, moldable thermoplastics or wet gauze commonly used in radiotherapy clinics.^7^ The manual process of matching the planned bolus on the treatment unit using these materials often leads to air gaps or bolus of varying thickness. The magnitude of these issues cannot be anticipated at the planning stage, and it is often assumed that day‐to‐day differences in bolus placement/density will blur out the deviations in dose delivery.

Any changes in fit were deemed to be due to changing patient anatomy as TPU has minimal shrinkage (0.4%–1.4%) which mainly occurs as the print cools.^27^ The TPU materials used in this study have a heat deflection temperature and melting point far greater than the ambient temperature when in use or during storage.^24^, ^25^, ^26^ Once printed, each bolus had no notable size or volume changes when compared on CBCT imaging to the original bolus contour.

The dosimetric consequences of air gaps have been well documented.^6^, ^20^, ^21^, ^22^, ^28^ Small air gaps under bolus material have been shown to produce only small decreases in surface and skin dose for photon fields, with at least 90%–94% of maximum dose still delivered with a 10 mm air gap between the bolus and the skin surface.^6^, ^28^ However, the effect of an air gap does also depend on field size, angle of incidence, and beam energy. For example, a small 5 × 5 cm field with an air gap of at least 5 mm produced a significant reduction in surface dose, which was insignificant for a 10 × 10 cm field until the air gap became >2 cm.^28^ A number of studies have evaluated the use of 3D printed boluses on phantoms or small samples of patients.^10^, ^12^, ^13^, ^18^, ^19^, ^29^ However, few investigated the application of flexible 3D printed bolus materials on a large cohort of clinical cases. For example, one study by Zhao et al.^12^ demonstrated the versatility of 3D printing in radiotherapy by describing three applications of 3D printing in radiotherapy; a photon bolus case, a modulated electron radiotherapy bolus case, and a High Dose Rate surface brachytherapy case. We evaluated its use on a large cohort in the form of a retrospective analysis.

We found that 3D printed bolus using TPU generally matched the planned bolus position with minimal air gaps of clinical significance for H&N, scalp, and extremity treatment sites. Each 3D printed bolus also tended to remain consistent over the course of treatment when evaluated in situ using CBCT imaging. It was found to be better suited for H&N, scalp, and extremity treatment sites, compared to the pelvic region. Due to the variability found using 3D printed bolus in the pelvis region, a 3D printed bolus approach is not currently undertaken in our clinic for this region. However, advancements in the 3D printing tools available now offer the possibility of printing a “mold” to create a poured silicone bolus that would have comparable flexibility of traditional bolus but also be customized to the patient anatomy.^30^ The evaluation of custom molds is outside the scope of this study and is for future investigation. For individual cases where a bolus is found to have a poor fit, a dosimetric evaluation is undertaken using our TPS. If the air gap results in a significant reduction in cover, a new bolus is drawn using the CBCT body contour and 3D printed as soon as possible.

Care is required when generating the bolus as any discrepancies in the patient's body contour, such as not accounting for the thermoplastic mask shell, at the planning stage will result in these errors being propagated into the bolus when printed. These issues may result in the bolus not fitting the patient appropriately, causing discomfort or generating air gaps between the bolus and the patient's skin surface. Changing patient contours due to edema, tumor response, or weight loss may also result in a change in the bolus fit and the need for a new bolus to be generated. Three‐dimensional printed bolus was most effective in cases where the CT body contour was easily defined and no contour changes were expected. For H&N cases, the thermoplastic shell offers a rigid surface and the fit was typically very consistent over the course of treatment. For cases where bolus was required to fit around complex external contours (e.g., H&N and extremity cases) a 1–2 mm uniform expansion of the body contour before bolus generation was undertaken in eclipse as standard. Presumably this corrected for small systematic differences between the automatic body contour generation in eclipse versus the true body contour resulting in a more “true” fit. Pelvic cases did not benefit from this extra expansion of the body contour before bolus generation, as fit issues were typically the result of variable external anatomy day‐to‐day.

Overall, treatment staff reported that the 3D printed bolus was easily and quickly placed on a day‐to‐day basis and was preferred to using sheets of uniform bolus, thermoplastics, and especially wet gauze.

## 5 CONCLUSION

This retrospective evaluation demonstrates that the use of 3D printed bolus, created with semi‐flexible printer materials, is an effective and practical choice for a number of treatment sites. Using a 3D printed approach for H&N, scalp, and extremities was found to have an excellent fit. Three‐dimensional printed bolus for pelvic cases was found to have the highest “acceptable” and “poor” ratings. The quality of fit was maintained throughout treatment for all cohorts except for the pelvis treatment sites which tended to have the highest change in quality of fit. Overall, a 3D printed approach for H&N, scalp, and extremity bolus has been well received in our clinic due to its ease of placement and consistent fit.

## AUTHOR CONTRIBUTIONS

Ciaran Malone was involved in the coordination of the study, undertook the statistical analysis and took lead on the study write‐up and data collection. Elaine Gill, Tanith Lott, Catherine Rogerson, Sinead Keogh, and Majed Mousli collaborated with lead author on initial study design, data collection, statistical analysis. John Gaffney provided clinical input from a radiation oncology perspective and undertook data collection. Denise Carroll and Caitriona Kelly collaborated with lead author on the initial setup of the 3D printing workflow used in this study and undertook independent validation of ratings for treatment fractions that were not in agreement after initial data collection. Brendan McClean assisted the lead author in the statistical analysis of data collected and the subsequent write‐up. All authors provided critical feedback and helped shape the research, analysis, and manuscript.

## CONFLICT OF INTEREST

Ciaran Malone has received a travel grant from Adaptiiv to present at ASTRO 2018 on the use of 3D printing in his department prior to this work being undertaken.

## Data Availability

Research data are stored in an institutional repository and will be shared upon request to the corresponding author.
